# Not forgetting the humanitarian contexts in the fight against antimicrobial resistance: Operational-driven reflection on knowledge and research gaps by Médecins Sans Frontières

**DOI:** 10.1371/journal.pgph.0005498

**Published:** 2025-12-11

**Authors:** Pilar Garcia-Vello, Carine Naim, Celine Langendorf, Clare Shortall, Dušan Jasovský, Fabiola Gordillo Gomez, Dea Abi Hanna, Mohamad Khalife, Aniroda Broomand, Jasper Wagan, Marie Poupard, Ismael Adjaho, Amrish Baidjoe, Rupa Kanapathipillai, Anna Farra

**Affiliations:** 1 Luxembourg Operational Research and Epidemiology Support Unit (LuxOR), Médecins Sans Frontières, Operational Centre of Brussels (OCB), Luxembourg, Luxembourg; 2 Middle East Medical Unit (MEMU), Médecins Sans Frontières, Operational Centre of Brussels (OCB), Beirut, Lebanon; 3 Medical Department, Médecins Sans Frontières, Operational Centre of Brussels (OCB), Brussels, Belgium; 4 Epicentre, Paris, France; 5 Manson Unit, Médecins Sans Frontières, Frontières Operational Center of Amsterdam (OCA), London, United Kingdom; 6 MSF Access, Médecins Sans Frontières International, Geneva, Switzerland; 7 Médecins Sans Frontières, Operational Centre of Geneva (OCG), Amman, Jordan; 8 Médecins Sans Frontières, Operational Centre of Amsterdam (OCA), Amsterdam, The Netherlands; 9 Médecins Sans Frontières, Operational Centre of Paris (OCP), Paris, France; 10 Médecins Sans Frontières, Operational Centre of Barcelona-Athens (OCBA), Barcelona, Spain; 11 Médecins Sans Frontières, West and Central Africa (WaCA), Abidjan, Côte d’Ivoire; 12 Médecins Sans Frontières, Operational Centre of Paris (OCP), New York, New York, United States of America; Aga Khan University, PAKISTAN

## Abstract

Despite Antimicrobial Resistance (AMR) being a growing threat to global public health, there is scarce evidence from humanitarian settings. Working in fragile contexts, Médecins Sans Frontières (MSF) is well positioned to identify knowledge gaps, raise unrecognised issues, and contribute to the global AMR agenda. Based on MSF’s experience, this review intends to highlight the research priorities on AMR for the most vulnerable patients. Infection Prevention and Control (IPC), Antimicrobial Stewardship (AMS), and Diagnostics and Surveillance (D&S) should be enhanced by sustainable and context-adapted approaches, especially by strengthening data collection and surveillance. Safety and efficacy studies focusing on vulnerable populations and the development of REASSURED diagnostics should be prioritised. Building evidence to address affordability and availability barriers of antimicrobials, vaccines, and diagnostics is equally essential. Vulnerable populations, including neonates, malnourished children, individuals living with advanced HIV, and patients with war-related injuries, must be placed at the centre of research agendas. It is also essential to address the intersections between AMR, climate change, and conflict. These research priorities are essential to preserving antimicrobial effectiveness and improving patient outcomes in the most neglected settings.

## Introduction

Antimicrobial resistance (AMR) is a growing serious threat to global public health associated with 4.95 million deaths, including 1.27 million attributable deaths in 2019 [1]. Projections estimate that AMR could translate into 169 million deaths, including 39.1 million attributable deaths between 2025 and 2050 [2]. The highest burdens are in sub-Saharan Africa and South Asia [1,2]. However, by providing better treatment for severe infections and improving access to antibiotics, 92 million of these deaths could be averted [1,2]. Beyond bacterial resistance, viral, fungal, and protozoal AMR also significantly contribute to the global health burden [3–5].

These disparities are further exacerbated by conflict, displacement, and climate change, which together erode health systems and the ability to respond to AMR [6–8]. Moreover, limited access to quality vaccines, antimicrobials and diagnostics in humanitarian settings exacerbates this imbalance, leading to suboptimal patient outcomes [9,10]. However, only about 10% of health research resources are devoted to addressing problems responsible for more than 90% of the global disease burden, the so-called 90/10 research gap [11].

In this critical landscape, Médecins Sans Frontières (MSF, Doctors Without Borders) plays a pivotal role by operating in some of the world’s most underserved regions. MSF focuses on the needs of the most vulnerable patients, including neonates, malnourished children, individuals living with advanced HIV, and those suffering from trauma and war-related injuries [12]. Other organisations, such as the International Committee of the Red Cross (ICRC), ReAct, the International Rescue Committee (IRC), or the Fleming Fund, also recognise the vulnerability of these groups to AMR [13–17].

Moreover, MSF has a unique capacity to conduct research in neglected contexts that would otherwise be inaccessible. It must constantly evaluate rapidly evolving situations to provide the best quality of care and ensure patient safety [18]. Therefore, optimising interventions to ensure antimicrobials remain as effective as possible and securing accessibility to the right antibiotics and quality diagnostics at the right time for all patients is key.

Despite the global recognition of the AMR crisis, research agendas remain inadequately aligned with humanitarian contexts. The recently published Global Research Agenda for AMR in Human Health by the World Health Organisation (WHO) [19] is a first necessary step to outlining research priorities. However, it could benefit from further consideration of the unique challenges in humanitarian settings. Investing in a better understanding of AMR in conflict zones, disaster areas, and regions with fragile healthcare systems is key to maintaining medical interventions. This is especially important considering that 2 billion people, a quarter of humanity, are estimated to live in conflict-affected contexts [20] and 4.5 billion people, more than half of the global population, face high risks from extreme weather, with 2.3 billion living in poverty [8].

This review, therefore, builds on MSF’s experience from over 50 AMR projects in over 20 countries and expert insights to identify global knowledge voids, raise previously unrecognised issues, and contribute to the international AMR agenda.

## Methods

This exercise was developed through a consultative narrative review with MSF working groups and referents and a comprehensive literature review. A total of 40 project and headquarters experts were consulted through workshops and multiple rounds of consultation. The literature review focused on peer-reviewed and grey literature published between 2018 and 2024 on PubMed, Google Scholar, and institutional databases. It aligns with the MSF strategy and the global priorities highlighted by the WHO. Tuberculosis, HIV and hepatitis B and C, mosquito-borne diseases (e.g., malaria, Zika virus, West Nile fever), neglected tropical diseases (NTDs), haemorrhagic diseases (e.g., Ebola, Marburg, Lassa and yellow fevers) and vaccination are covered by other medical units. Nonetheless, the MSF AMR agenda allows for interaction between portfolios. In addition, the inclusion of certain topics in this exercise is primarily driven by the significant threat they pose to vulnerable populations, including HIV and tuberculosis patients.

## Results

**Fig 1:**
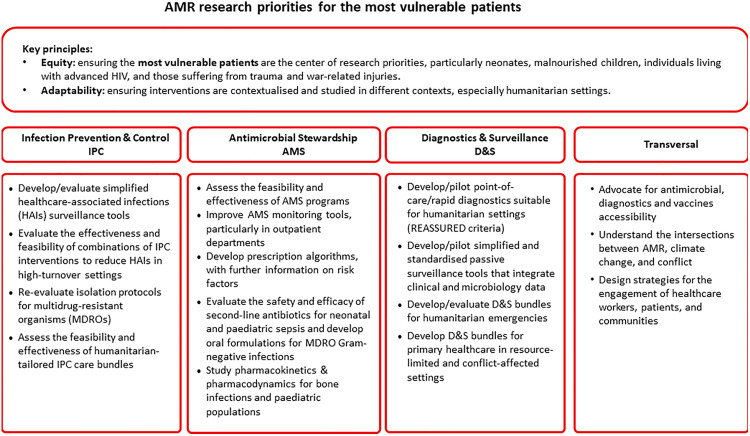
AMR research priorities for the most vulnerable patients that should be at the centre of global research agendas.

The structure of this exercise follows the key domains Infection Prevention and Control (IPC), Antimicrobial Stewardship (AMS), Diagnostics and Surveillance (D&S), and transversal research presented in approximate order of clinical and operational priority to provide an overview of the challenges faced in humanitarian settings (Fig 1).

### Infection prevention and control (IPC): Addressing healthcare-associated infections

Infection prevention and control (IPC) is fundamental to all patient care and health interventions. Recent estimates suggest that in low- and middle-income countries (LMICs), at least 337,000 AMR-associated deaths could be prevented yearly through the combination of known interventions, including IPC [21]. Universal access to high-quality Water, Sanitation and Hygiene (WASH) facilities could prevent a further 247,800 deaths, and paediatric vaccines another 181,500 [21]. In humanitarian settings, key barriers to effective IPC implementation include poor infrastructure, which in turn limits isolation capacity, often results in overcrowding, which prevents sufficient bed spacing, and the absence of pragmatic surveillance systems that allow for earlier detection of AMR-related nosocomial outbreaks. These structural constraints hinder the consistent application and monitoring of IPC measures across health facilities.

#### Bridging evidence gaps to prevent and control healthcare-associated infections (HAIs).

Healthcare-associated infections (HAIs) are a major source of morbidity, mortality, and AMR drivers [22]. Despite its importance, evidence on the effectiveness and cost-efficiency of specific IPC interventions, such as cohorting, isolation practices, staffing ratios, and the presence of IPC focal points, remains limited, especially in humanitarian settings. Tools and strategies showing cost-effectiveness in high-income countries often cannot be used in humanitarian settings due to a lack of data [23]. This underscores the urgent need for pragmatic research to evaluate combinations of IPC interventions under real-world conditions [22,24–29]. While the WHO’s prioritisation exercise reinforces the central role of IPC globally, further operational guidance tailored to the specific constraints of humanitarian settings would help translate global recommendations into field-level impact [19].

Likewise, simplified, standardised surveillance tools to monitor HAIs across formal and informal health systems are urgently needed. Monitoring IPC adherence through audits and feedback is essential for preventing and managing HAIs and outbreaks [29,30]. Establishing surveillance systems, including risk-based outbreak prediction algorithms, can greatly enhance preparedness. Developing and evaluating context-adapted monitoring tools and indicators is critical to optimise data collection and reporting, particularly outside formal health systems such as refugee camps. Moreover, although WHO supports microbiology-informed surveillance, it could benefit from further guidance on when and how to implement laboratory-based surveillance in humanitarian settings. Additionally, the lack of reliable patient tracking systems further complicates surveillance efforts in these environments. As such, IPC-informed surveillance must be carefully prioritised and supported through capacity building, infrastructure investments, and practical implementation strategies.

#### Re-evaluate isolation protocols for multidrug-resistant organisms (MDROs).

In humanitarian settings, isolation capacity is often limited or absent. Moreover, there is limited information on the community-based transmission of numerous multidrug-resistant organisms (MDROs) in many regions of the world [31]. The rationale for isolation protocols should be re-evaluated critically, particularly in settings with high community transmission of specific MDROs. Clear guidance should be established for when isolation measures can be modified or discontinued, based on local epidemiology and feasibility.

As mentioned in the WHO agenda, isolation of MDRO-infected or colonised patients, decolonisation of healthcare workers, and routine catheter changes are three areas that could greatly impact the incidence of HAIs [19]. However, operational clarity is needed on when and how to apply these measures, especially in crisis contexts. Guidelines must also be adapted to high surgical volumes and vulnerable populations, including neonates, malnourished children, people living with HIV, and those with war-related injuries.

#### Assess the feasibility and effectiveness of humanitarian-tailored IPC care bundles.

A range of IPC care bundles have been recommended, yet in humanitarian settings, the evidence base guiding which bundles are most feasible and effective remains thin [32]. Operational guidance on how to adapt and prioritise these bundles in resource-limited and conflict-affected settings is limited [32]. In acute crisis contexts, there is little clarity on which bundle components are most impactful or how to adapt existing bundles to local constraints. There is a pressing need for humanitarian-specific operational evidence to inform global IPC standards and context-adapted standard operating procedures (SOPs). Surveillance systems should be aligned with implementable guidelines; this way, humanitarian actors could build more resilient and effective IPC programmes.

MSF’s experience highlights the need for longitudinal research to assess the long-term impact and sustainability of IPC strategies. Therefore, IPC research must focus on practical, context-adapted interventions instead of idealised frameworks. Supporting the reduction of structural barriers, behaviour change, leadership in daily practice, and institutional accountability for IPC is a priority.

### Antimicrobial stewardship (AMS): Rationalising antibiotic use

Antimicrobial stewardship (AMS) aims to safeguard the effectiveness of antimicrobials and patients’ safety. Misuse of antimicrobials in human health, animal husbandry, and agriculture accelerates the development and spread of AMR [33]. Therefore, the rational use of antibiotics is not just a matter of good practice, it is a fundamental ethical responsibility. Implementing AMS programmes in fragile settings requires different approaches and identifying the challenges and facilitators of AMS program implementation is crucial to developing efficient and sustainable AMS strategies.

#### Assess the feasibility and effectiveness of AMS programmes.

Evidence on the cost-effectiveness of AMS interventions and how significantly these interventions reduce the burden of AMR and reduce patient mortality and morbidity primarily comes from high-income settings [34,35], however, evidence from LMIC is scarce [21]. Research is needed in humanitarian settings where resources are limited, and AMR interventions can be perceived as an unnecessary cost and burden. External factors, including non-bacterial outbreaks (e.g., respiratory viruses, malaria, scabies), climate change, and conflict, may indirectly influence antimicrobial prescribing practices and resistance trends [7,36–39]. Further evaluation of these drivers is necessary to inform AMS strategies adapted to dynamic and fragile contexts.

#### Improve AMS monitoring tools.

A significant challenge in AMS implementation is the lack of accessible, user-friendly tools to monitor antimicrobial use and consumption, clinical decision-making, and stock availability, especially in settings with fragmented information systems [19]. Developing simplified, robust, and standardised monitoring tools is essential to track the progress of AMS programmes, including antimicrobial use, consumption, access and shortages. The feasibility and acceptability of these tools should be assessed following implementation.

Special attention must be given to paediatric antimicrobial use, as traditional Defined Daily Dose (DDD) metrics are poorly suited for children and neonates [40,41]. Therefore, alternative user-friendly and adaptable measurement approaches, coupled with learning and development (L&D) initiatives for healthcare workers (including pharmacists), are needed to accurately capture paediatric and neonatal prescribing, particularly in outpatient departments [2]. Lack of systems to routinely collect patient-level data to monitor antimicrobial use remains a major limitation regarding AMS in LMIC – periodic audits of patient files are necessary to have visibility on prescribing practices. Integrating stockout data in AMS monitoring could provide valuable information on the antibiotic access gaps and prescribing shifts driven by medication shortages.

#### Increase evidence for guidelines, including prescription algorithms.

A critical need remains for the development of tailored prescription algorithms for settings with limited access to medicine, diagnostics and healthcare. WHO has highlighted the importance of investigating criteria and strategies to optimise empirical antimicrobial therapy for the main infectious syndromes, particularly in such settings [19]. These should incorporate patient risk factors and biomarkers [42,43]. Evidence is required to identify the nuanced contribution of malnutrition, malaria, tuberculosis, and HIV as risk factors for particular MDROs, which could be further explored in the WHO agenda [19,44]. Additionally, integrating electronic AMS systems implementable in humanitarian settings could further support these efforts [45,46].

Local resistance patterns should guide empirical guideline development, though the availability of quality-assured microbiology results is scarce in LMIC. Sentinel laboratories should be identified and supported for quality assurance; moreover, microbiological data from these sites should be linked to clinical information to enable the meaningful interpretation of local cumulative antibiograms. Alternatively, point prevalence surveys assessing bacterial causes of sepsis should be performed and the results published to inform clinical practice. In parallel, research is needed to define resistance thresholds that should trigger updates to local empirical regimens and changes in IPC strategies.

#### Evaluate the safety and efficacy of second-line antibiotics for neonatal and paediatric sepsis and develop oral formulations for multidrug-resistant Gram-negative infections.

As recognised by WHO, access to appropriate second-line antibiotics remains limited in many humanitarian settings [19,47]. Clinical trials on paediatric sepsis are needed to evaluate the safety and efficacy of second-line regimens, such as carbapenem-sparing combinations (e.g., ceftriaxone-amikacin or piperacillin-tazobactam). Moreover, the development of oral formulations for multidrug-resistant Gram-negative infections is urgently needed [19,48]. Oral treatment options would immensely benefit patients, especially when prolonged courses of treatment are required or when intravenous therapy is not possible due to infrastructure or staffing constraints.

#### Study pharmacokinetics and pharmacodynamics (PK/PD) for bone infections and paediatric populations.

There is a critical lack of data on the optimal dosing of antimicrobials in paediatric and malnourished populations, as well as for deep-tissue and bone infections [19]. MSF calls for research to focus on conducting PK/PD studies tailored to these vulnerable groups, allowing patients to access a safe and effective dosing regimen.

Pharmacovigilance systems must also be strengthened to monitor, report, and prevent issues related to antimicrobial quality, safety, and misuse [49]. These systems are especially critical for antimicrobials associated with significant side effects (e.g., glycopeptides and aminoglycosides) and antimicrobials without solid evidence for their use in neonates and children. Moreover, simplified, point-of-care (POC) Therapeutic Drug Monitoring (TDM) could greatly improve patient outcomes in humanitarian settings [50,51].

### Diagnostics and Surveillance (D&S): Filling global blind spots

Diagnostics and surveillance (D&S) are fundamental to guide treatment and monitor resistance trends. D&S ensure patients receive the antibiotics they need, supports the health system in preparing for and responding to ongoing challenges, and supports the detection, identification and management of outbreaks [3,52,53]. D&S capacity around the world is suboptimal [54]. MSF and other actors are playing a role in increasing accessibility to bacteriology laboratories around the world, as well as increasing diagnostics accessibility and quality through long-term capacity building, training the local laboratory staff on updated guidelines for bacteriology procedures and with innovative initiatives such as the Mini-Lab and Antibiogo [55,56].

#### Develop/pilot point-of-care/rapid diagnostics suitable for humanitarian settings (REASSURED criteria).

Currently, the diagnostic tools available in resource-limited settings provide pathogen identification and susceptibility testing in 48–72 hours versus 4–12 hours in high-income countries. More research is needed to provide diagnostic tools based on REASSURED criteria: real-time connectivity, ease of specimen collection, affordable, sensitive, specific, user-friendly, rapid, equipment-free, and delivered to be suitable for field use in humanitarian contexts [57]. Global efforts should aim to validate and introduce diagnostic tools that meet these criteria as much as possible, particularly for syndromes like fever of unknown origin, childhood pneumonia, or suspected neonatal sepsis, in areas where diagnostic uncertainty leads to inappropriate antibiotic use. POC C-reactive protein (CRP) and procalcitonin tests, ultrasound (POCUS), and computer-aided detection (CAD) algorithms may play a crucial role, and clinical algorithms should be developed, including the use and interpretation of these POC diagnostic tests, and their impact assessed post-implementation [58].

#### Develop/pilot simplified and standardised passive surveillance tools that integrate clinical and microbiology data.

AMR surveillance data is typically generated through passive surveillance, which involves bacterial isolates from clinical samples of patients with suspected infections. Passive or health facility-level surveillance is the basis for monitoring resistance trends, improving empiric treatments, addressing critical gaps, and enhancing the global response. While the WHO mentions strengthening surveillance, it could benefit from specific guidance on harmonising systems or the need to increase diagnostics capacity sustainably and cost-effectively [19]. Moreover, the link between clinical and microbiological data is rarely achieved in humanitarian settings.

Existing platforms like WHONET offer analysis for microbiology data with a focus on AMR surveillance but have some limitations. For example, it lacks integration with the clinical information from electronic health record systems [59]. Limitations of WHONET should be explored and jointly addressed, for example, the integration of other health data systems or further variables. Moreover, strengthening the link between WHONET and advanced statistical tools and algorithms will facilitate more robust data analysis and improve evidence-based decision-making [60,61]. Laboratory standardisation and validation, as well as data-sharing networks, should be strengthened.

#### Develop/evaluate contextualised and rapidly deployable D&S bundles for humanitarian emergencies and primary healthcare in humanitarian settings.

Further research is needed to strengthen the evidence on the impact and efficiency of D&S interventions, as well as the implementation factors that influence their success. This evidence is essential to inform guideline updates and to define and prioritise essential diagnostic packages required for all three levels of healthcare: primary care, primary care with basic laboratory, and hospitals [52,62]. Guidelines that define the criteria to decide whether to implement a conventional laboratory or alternatives, such as the Mini-Lab, to ensure context-appropriate diagnostic capabilities.

Although the deployment of the Mini-Lab is faster than conventional laboratories, it still requires training the staff on sample collection and processing, as well as results interpretation. Moreover, in many settings, microbiology certification is not part of university curricula [56]. Further investment in mobile and adaptable microbiology solutions, including trained staff, is needed to support faster deployment in emergencies. These microbiology diagnostics solutions should integrate a pool of microbiology-trained staff, essential POC diagnostics and prioritise the identification and resistance profiling of high-risk pathogens among war-injured patients, where infections with MDROs are particularly concerning.

### Transversal operational research on AMR

Operational research should extend beyond technical solutions to tackle underlying drivers of AMR, such as conflict, climate change and natural disasters and examine systemic barriers to IPC, AMS and D&S. As well as the roles that gender, displacement and social determinants of health play in accessibility to mitigation measures. Other critical areas include the effectiveness of different modalities in community engagement, healthcare personnel empowerment, health promotion, and advocacy in addressing AMR.

#### Research and development of vaccines, antimicrobials and diagnostics – Accessibility.

While the WHO AMR research agenda highlights the need for developing new antimicrobials, vaccines, and diagnostics, MSF emphasises the equally critical need to ensure the accessibility—availability, affordability, and practicality—of existing tools in humanitarian settings [2,9,10,19,63]. Stockouts, supply chain disruptions, and lack of affordability continue to hinder appropriate use, impact clinical decision-making, and contribute to the spread of AMR. Research must prioritise understanding the link between access barriers and patient outcomes and developing solutions such as supranational platforms for pooled procurement, geographically diversified manufacturing, and strengthening forecasting and supply chain management.

The innovation ecosystem should also ensure that the needs of LMICs are integrated from discovery through implementation. Public and non-profit R&D efforts should be supported, as these are most conducive to access, affordability, stewardship, and the kind of collaboration (such as sizeable global clinical trial networks with sites in LMICs) that is needed to overcome the scientific bottlenecks in antibiotic R&D. R&D prioritisation must be public‑health driven, ensuring that medicines and diagnostics are adapted for fragile contexts. For example, favouring oral over intravenous formulations, with room‑temperature stability, simplified dosing, and providing child‑friendly options [63–66].

Further investment is needed in POC and in vitro diagnostics that meet REASSURED criteria and in the expansion of molecular tools like next-generation sequencing [19,57,63,65]. Comparative studies on feasibility, cost-effectiveness, and impact are essential to validate diagnostics in humanitarian contexts. Multi-disease platforms and integration with digital health systems could also improve surveillance and outbreak response. Ultimately, tailored, stable, and context-adapted tools are essential to support effective treatment and reduce AMR in the world’s most vulnerable populations.

#### Understand the intersections between AMR, climate change, and conflict.

Climate change and conflict are significant drivers of infectious diseases by disrupting healthcare, water and sanitation and food systems. More than half of the global population, 4.5 billion people, face high risks from extreme weather such as floods and droughts, with 2.3 billion living in poverty [8]. These issues, though mentioned in the WHO’s agenda, are even more important in humanitarian contexts [19]. Rising temperatures and extreme weather events have been linked to increased disease, such as vector-borne, waterborne and fungal infections [7,39,67]. Simultaneously, conflict weakens healthcare systems by damaging facilities, interrupting essential supply chains, and exhausting healthcare workforce capacity [68–70]. Food insecurity, exacerbated by climate-related crop failures and conflict-induced instability, increases the risk of malnutrition, which increases vulnerability to infections [6,39,71]. Moreover, climate change, natural disasters and conflict drive population displacement, which also increases the risk of AMR as these populations face unique barriers to accessing healthcare. A deeper understanding of prevalence, drivers and outcomes in these settings is essential to develop inclusive interventions addressing AMR and its drivers [72].

However, the nuances of the interaction of climate change and conflict with AMR are poorly understood, demanding targeted operational research to fill critical knowledge gaps and inform inclusive strategies.

Global AMR research agendas and policies predominantly focus on formal healthcare systems and may not fully account for conflict zones, internally displaced people/refugee camps, and climate change-affected settings [19]. This is especially significant considering 2 billion people, a quarter of humanity, are estimated to live in conflict-affected contexts [20]. The complex challenges of population displacement, erratic supply chains, etc., are barely mentioned in the agenda. Moreover, there is an urgent need for evidence and innovation that is adaptable to the realities of humanitarian settings.

#### Design strategies for the engagement of patients, communities, and healthcare workers.

There is limited evidence on the effectiveness of patient, their caregivers and community engagement initiatives in reducing AMR [73–75]. More research is needed to understand how patients, their caregivers and communities can be actively involved in IPC and AMS. The WHO’s agenda could benefit from a more structured approach to integrating patient, caregivers and community perspectives into AMR strategies, ensuring that interventions are not only scientifically sound but also socially and culturally relevant and therefore better meet contextual needs.

Healthcare workers also play a crucial role in IPC and AMS, yet existing programmes often lack comprehensive, evidence-informed, context-specific training, particularly in fragile settings. Medical doctors, nurses, social workers, infection control officers, health promoters, and pharmacists should be trained and involved in IPC and AMS programmes [76].

## Discussion and conclusion

AMR poses an existential threat to global health, yet research priorities often neglect the populations and contexts where the burden is greatest and the means to address AMR are most limited. MSF advocates for research agendas that prioritise equity, focusing on neonates, malnourished children, individuals living with advanced HIV, and patients suffering from war-related injuries, without forgetting women and the elderly, and adaptability, ensuring that interventions are designed for dynamic, fragile humanitarian settings.

Despite increasing international attention to AMR, the evidence base remains insufficient to drive localised action and resource mobilisation toward the benefit of patients in humanitarian settings [12]. MSF has made strides in undertaking operational research in vulnerable contexts, which are also the most impacted by AMR [1,2]. Despite these efforts, critical evidence gaps hinder effective interventions. Patients in humanitarian settings face context-specific barriers and challenges. Identifying these challenges and finding pragmatic contextualised solutions for these settings is key.

In IPC, overcrowding, limited water access, inadequate isolation capacity, and weak information systems are pervasive challenges. Research must move beyond describing these barriers to evaluating practical, scalable solutions. In particular, innovative HAI monitoring tools, such as real-time dashboards, risk scoring systems, and early outbreak detection algorithms, are needed to strengthen preparedness and resilience across settings.

In AMS, the lack of accessible metrics and tools to monitor antimicrobial consumption, prescribing patterns, and shortages or stockouts, particularly in settings with fragmented data systems and supply chains, is still a challenge. These gaps are particularly acute in outpatient settings, where consumption data is sparse [2]. Developing robust, simplified tools that also integrate stockout data would enhance understanding of antimicrobial misuse and access barriers. In parallel, tailored prescribing algorithms should be developed, and the safety, efficacy, and PK/PD of existing antibiotics should be evaluated, considering comorbidities such as malnutrition, malaria, tuberculosis, and HIV is essential to improve therapy in settings with limited capacity.

D&S remain underdeveloped in fragile settings. REASSURED POC, rapid, and in vitro diagnostics must be designed to enable D&S in these contexts. Contextual approaches are necessary to adjust algorithms and tools to fit our specific challenges, at patient and hospital levels, particularly in areas with limited microbiological data. Additionally, emergency microbiology bundles that could easily be deployed are required. These emergency/mobile labs should integrate essential POC diagnostics and prioritise high-risk pathogens, particularly among war-injured patients. Surveillance platforms that link clinical and microbiological data should be enhanced.

Finally, building evidence to advocate for the affordability and availability of antimicrobials, vaccines, and diagnostics is key. It is also important to consider the intersections between AMR, climate change, conflict, and population displacement in these studies. Above all, engaging patients, caregivers, communities, healthcare workers, and policymakers in these efforts is key to designing sustainable strategies.

The WHO Global Research Agenda for AMR in Human Health is a first necessary step to outlining research priorities [19]. However, it could have further integrated humanitarian priorities and emphasised vulnerable populations that are disproportionately affected by AMR. Indeed, malnutrition is underrepresented, particularly in the priority of paediatric sepsis.

The priorities highlighted in this passage and by WHO risk remaining an unfulfilled ambition if not supported by adequate technical and financial resources to address the evidence asymmetry between and among countries. The means to global health research are direly lacking, as only 10% of resources are allocated to address health issues in low-resource settings where 90% of the worldwide disease burden is concentrated [11]. This so-called 90/10 research gap is especially pronounced in the case of AMR. For example, fewer than 1.3% of 50,000 laboratories in Africa have the microbiology capacity to diagnose AMR [77]. Therefore, today’s available evidence does not represent the realities MSF witnesses in such contexts. MSF’s research was made possible through the establishment of conventional and Mini-Labs, the integration of Antibiogo, the training of AMS and IPC focal points, and the enhancement of information systems and epidemiological mentoring capacity. Accelerating research, understanding AMR in such contexts, and proposing effective intervention options adapted to such contexts require collaboration among several actors. The proposal of an Independent Panel for Action against AMR (IPEA) can play a vital role in identifying and mobilising resources toward addressing these knowledge gaps [78]. We emphasise the role of supporting and ensuring the participation of civil society organisations in the governance of such initiatives to balance evidence asymmetry and ensure an unbiased interpretation of evidence.

Advancing MSF’s research priorities on AMR is not only critical for improving patient outcomes in humanitarian settings but is also essential to safeguard the effectiveness of antimicrobials for all. Contextualised, equity-driven research must become the cornerstone of the global AMR response.
